# The Role of Kidney Function on the Biomarkers of Neurodegeneration and Cognitive Decline in Older Adults

**DOI:** 10.1002/alz70856_104081

**Published:** 2025-12-24

**Authors:** Anisa Dhana, Charles Decarli, Klodian Dhana, Pankaja Desai, Kyle Dennis, Denis A Evans, Kumar B Rajan

**Affiliations:** ^1^ Rush University Medical Center, Chicago, IL, USA; ^2^ University of California, Davis, Davis, CA, USA; ^3^ Rush University Medical Center, hicago, IL, USA; ^4^ Rush Institute for Healthy Aging, Chicago, IL, USA; ^5^ University of California Davis, Davis, CA, USA

## Abstract

**Background:**

Biomarkers of neurodegeneration–such as neurofilament light (NfL), total tau (t‐tau), and glial fibrillary acidic protein (GFAP)–have been associated with cognitive decline and the risk of Alzheimer's disease. However, their levels in serum may also be influenced by reduced kidney function, even in the absence of cognitive impairment. Therefore we aimed to evaluate the association of kidney function with serum biomarkers of neurodegeneration and, subsequently, to determine whether kidney impairment modifies the association of NfL, GFAP, and t‐tau with longitudinal changes in global cognition.

**Method:**

This study included 1207 individuals aged 65 years and older from the Chicago Health and Aging Project. Kidney function was determined based on the estimated glomerular filtration rate (eGFRcr) according to the 2021 Chronic Kidney Disease Epidemiology Collaboration and categorized into normal eGFRcr>=60, impaired eGFRcr=30‐59, and severe eGFRcr<30 mL/min/1.73m2. Biomarkers of neurodegeneration were measured in the serum by a bead‐based HD‐X molecular immunoassay platform. Global cognition was assessed using established cognitive tests. Multivariable adjusted linear and mixed‐effect regression models were used to evaluate the association between kidney function, neurodegeneration biomarkers, and cognitive decline.

**Results:**

Of 1,207 individuals included in the analysis, 738 (61.1%) were women, 716 (59.3%) were African American, and the mean (SD) age of the overall study sample was 78.4 (6.2) years. Lower levels of eGFRcr were associated with higher levels of NfL, GFAP, and t‐tau in the serum. Specifically, per 1‐SD decrease in eGFRcr levels in serum, the NfL increased by 22.1% (beta=0.087, SE=0.007, *p*‐value<0.001), GFAP by 8.0% (beta=0.034, SE=0.007, *p*‐value<0.001), and t‐tau by 6.3% (beta=0.027, SE=0.012, *p*‐value<0.025). In addition, compared to people with normal kidney function, those with impaired and severe kidney function had 24.0% and 146.7% higher levels of NfL, respectively. Kidney function was not associated with cognition and did not modify the association of NfL, GFAP, and t‐tau with the rate of cognitive decline [three‐way‐interactions (i.e., biomarkers * eGFRcr * time) was non‐significant (*p* >0.235)].

**Conclusion:**

In conclusion, this population‐based study suggests that impaired kidney function may affect the levels of neurodegeneration biomarkers, and therefore, eGFRcr should be considered when using NfL, GFAP, and t‐tau in research and clinical applications.

